# Quantifying the socio-economic impact of leg lymphoedema on patient caregivers in a lymphatic filariasis and podoconiosis co-endemic district of Ethiopia

**DOI:** 10.1371/journal.pntd.0008058

**Published:** 2020-03-03

**Authors:** Thais Caprioli, Sarah Martindale, Asrat Mengiste, Dereje Assefa, Fikre H/Kiros, Mossie Tamiru, Nebiyu Negussu, Mark Taylor, Hannah Betts, Louise A. Kelly-Hope

**Affiliations:** 1 Department of Tropical Disease Biology, Centre for Neglected Tropical Diseases, Liverpool School of Tropical Medicine, Liverpool, United Kingdom; 2 National Podoconiosis Action Network, Addis Ababa, Ethiopia; 3 Federal Ministry of Health, Addis Ababa, Ethiopia; University of Washington, UNITED STATES

## Abstract

**Background:**

Lymphoedema caused by lymphatic filariasis (LF) or podoconiosis can result in physical disability and social exclusion, which is exacerbated by painful acute dermatolymphangioadenitis (ADLA) episodes. These conditions have a significant impact on patients, however, little is known about the indirect effects on their caregivers. This study, therefore, aimed to determine the impact on caregivers for patients with leg lymphoedema in a co-endemic district of Ethiopia.

**Methodology/Principal findings:**

A cross-sectional survey of lymphoedema patients and their caregivers was conducted using semi-structured questionnaires in the Southern Nation Nationalities Peoples Region (SNNPR) of Ethiopia. Lymphoedema patient information on clinical severity (mild, moderate, severe), frequency of ADLAs, their socio-demographic characteristics and the identity of main caregiver(s) was collected. Caregiver information on socio-demographic characteristics, types of care provided, their quality of life (QoL) measured across nine domains, and productivity was collected, with key indicators compared in the presence and absence of patients’ ADLAs.

A total of 73 patients and 76 caregivers were included. Patients were grouped by mild/moderate (n = 42, 57.5%) or severe (n = 31, 42.5%) lymphoedema, and reported an average of 6.1 (CI± 2.18) and 9.8 (CI± 3.17) ADLAs respectively in the last six months. A total of 48 (65.8%) female and 25 (34.2%) male patients were interviewed. Caregivers were predominately male (n = 45, 59.2%), and spouses formed the largest caregiving group for both female and male patients.

In the absence of an ADLA, most caregivers (n = 42, 55.2%) did not provide care, but only one caregiver did not provide care during an ADLA. In the absence of an ADLA, the average time (hour:minute) spent by mild/moderate (00:17, CI: ± 00:08) and severe (00:10, CI: ± 00:07) patient caregiver per task was minimal. The time mild/moderate (00:47, CI: ± 00:11) and severe (00:51, CI: ± 00:16) patient caregivers spent per task significantly increased in the presence of an ADLA. In addition, caregivers’ QoL was negatively impacted when patients experienced an ALDA, and they had to forfeit an average of 6 to 7 work/school days per month.

**Conclusion/Significance:**

Lymphoedema and ADLAs impact negatively on patients’ and their caregivers’ lives. This emphasises the importance of increasing access to effective morbidity management and disability prevention services to reduce the burden and help to address the Sustainable Development Goal (SDG) 5, target 5.4, which seeks to recognise and value unpaid care and domestic work.

## Author summary

Lymphatic filariasis and podoconiosis can result in chronic severe lymphoedema of the limbs, which is aggravated by frequent painful episodes of acute bacterial infection known as ‘acute attacks’. The debilitating impact of these conditions on patients are well documented, however, the extent to which the hardship extends to their caregivers is unknown. To address this knowledge gap, a survey of 73 lymphoedema patients and their caregivers was conducted in an endemic region of Ethiopia. Patients were found to be predominately female and caregivers predominately male, mostly spouses. Caregivers provided a wide range of care, and the type, frequency and time spent on tasks significantly increased when patients experienced an acute attack. Caregivers reported a negative impact on their quality of life and provided care for an average of 15 days and forfeited an average of 7 days of work or school per month. This study has revealed a previously hidden burden of lymphoedema that not only affects patients, but their caregivers who are mostly family members. This emphasises the importance of increasing access to effective morbidity management and disability prevention programmes to reduce the burden and help to address the Sustainable Development Goal (SDG) 5, target 5.4, which seeks to recognise and value unpaid and domestic work.

## Introduction

Lymphatic filariasis (LF) and podoconiosis are chronic disabling neglected tropical diseases (NTDs) that affect millions of people worldwide [1]. LF is a mosquito-borne disease caused by the parasites *Wuchereria bancrofti*, *Brugia malayi* and *Brugia timori* which are endemic in 73 countries with an estimated 40 million people affected clinically with lymphoedema and hydrocoele [2]. Infections are usually acquired during childhood, but the physical impairments develop later in life due to a dysfunctional and damaged lymphatic system, which causes fluid accumulation in the limbs, breasts and genitalia [3]. Several stages of limb lymphoedema exist, and whilst mild stages are reversible overnight, severe stages are permanent and can result in significant disability and loss of independence [3–5]. Alongside the disfigurements, patients experience re-current acute dermatolymphangioadenitis (ADLA) known as ‘acute attacks’, which are episodes of febrile illness, malaise and extensive swelling caused by secondary bacterial infection [6,7]. ADLAs have been identified as a critical factor contributing to the progression of diseases and predictor of disability [8–10]. Productive hours are lost, and the impairments render a socially excluded reality for many, thereby, contributing to the global loss of 2.78 million Disability Adjusted Life Years (DALYS) [11].

Podoconiosis is largely concentrated in Africa, and is known or suspected to be endemic in 32 countries affecting an estimated four million people worldwide [12–14]. Unlike LF, podoconiosis is a non-communicable disease, which is caused by the continuous exposure to irritant soil in barefoot populations [15,16]. Penetrated soil particles initiate the inflammatory cascade, which hinders the lymphatic system and results in the progressive bilateral swelling of the lower limbs. Similar to LF, several stages of lymphoedema exist [4], and the most severe stages are associated with frequent ADLAs and increased disability. The incapacitating and stigmatised impairments fuels severe economic consequences, inter-generational poverty [17] and alienates patients from society, including school, community meetings, churches and mosques [18].

The disability posed by leg lymphoedema, regardless of the aetiology, is likely to extend beyond the patient, to their caregivers. The World Health Organization (WHO) [19] defines a caregiver as: *‘A person who provides support… with various activities to persons with disabilities or long-term conditions… (who) provides emotional or financial support*, *as well as hands-on help with different tasks’*. Caregivers in lower middle-income countries (LMIC) have been characterised as poorly educated middle-aged wives [20]. Although caregiving may be largely rewarding, it can be laden with physical and emotional adversities, collectively known as the ‘caregiving burden’ [21]. Straining duties and instilling the obligation to accomplish all roles, caregiving sustains an incremental build-up of stress [20,22]. Caregiving is time consuming, productive hours are forfeited and caregivers’ psychological health is known to suffer [20,23,24]. Furthermore, cognisant of the degree to which the social pathology of the stigmatised impairments afflicts lymphoedema patients [25,26], whether their caregivers suffer additional hardship has yet to be ascertained.

Overall, there is little research on caregivers of people affected by NTDs. It has been estimated that five million LF patients are reliant on caregivers [27]. For limb lymphoedema, the type and frequency of care required is likely to be affected by the severity and the occurrence of ADLAs [28–30]. During ADLAs, patients are likely to rely on greater assistance to perform self-care and day-to-day activities due to their reduced mobility. This was reported in a recent paper which qualitatively outlined the financial and emotional worries instilled in podoconiosis caregivers whilst caring for patients during their ADLAs [31]. Furthermore, the gender of the caregivers can influence the types of care provided [26], women may be more likely to assist in personal care, than men [24]. Understanding the gender roles and it’s contributions to the hidden work caregiving embodies is important [32] as it is likely to pose a significant socio-economic burden on families and communities [33]. It is also fundamental to ensuring gender equity, which has been explored in relation to NTD mass drug administration (MDA) programmes [34]. Further, it may help achieve the Sustainable Development Goal (SDG) in relation to NTDs [35], as well as address SDG 5, target 5.4, which seeks to recognise and value unpaid care and domestic work [36].

LF and podoconiosis morbidity management and disability prevention (MMDP) programmes largely consist of limb hygiene and self-care activities, which are paramount to alleviate suffering and to reduce the occurrence of ADLAs [37]. It is pragmatic, cost-effective and efficient to integrate the LF and podoconiosis MMDP programmes where possible [38]. Involving caregivers may optimise the use of human resources of health, encourage the continuity of care and promote societal disability inclusion. In settings where caregivers form the linchpin of informal care, their welfare and, indirectly, their ability to provide care, is pivotal. Hence, a better understanding of who these caregivers are—how they assist- and what impact caregiving has on their quality of life (QoL), needs to be established.

This study, therefore, aimed to determine the socio-economic impact on caregivers for patients with leg lymphoedema in Ethiopia, where an estimated 5.6 million individuals are at risk of LF and 34.9 million individuals are at risk of podoconiosis [38]. A cross-sectional survey in a co-endemic district highlighted caregiver socio-demographic characteristics, types of care provided, QoL, and productivity, with key indicators compared in the presence and absence of patients’ ADLAs.

## Methods

### Ethics statement

Ethical approval was obtained from the Southern Nations Nationalities Peoples Region (SNNPR) Health Bureau and the Research Ethics Committee of the Liverpool School of Tropical Medicine prior the commencement of the study. All survey participants participated on a voluntary basis and informed consent was obtained. Participants who were literate provided written informed consent, and those who were illiterate had the information sheet read to them and oral informed consent was documented. For those aged <18 years, parental or guarantor informed consent was obtained, and they were present during the survey interview.

### Study site and characteristics

The study was conducted in the South Ari district of the South Omo Zone, within the SNNPR. The South Ari district comprises of 50 kebeles (villages) and has an estimated population of 265,866 people [39]. In 2015, using a probability-based sampling of school attending children to determine the prevalence of LF antigenemia, the South Ari district was re-mapped for LF. Results showed that 2.1% of immunochromatographic tests (ICTs) were positive, and the district was recommended to undergo MDA [40]. For podoconiosis, it is reported that the SNNPR has a prevalence of 8.63% (Confidence Interval (CI) ± 0.66%) [41].

Information on the burden of leg lymphoedema was obtained from a community-based integrated morbidity mapping survey conducted in 2015 where the severity of lymphoedema, caused by LF or podoconiosis, was staged as mild, moderate or severe using WHO guidelines [42]. The South Ari district recorded 2,277 lymphoedema patients with mild (n = 1,588, 66.8%), moderate (n = 655, 27.5%) or severe (n = 135, 5.7%) conditions [42]. The Gazer and Metzer health catchment areas were selected for the current study due to the high number of lymphoedema patients. Gazer health catchment recorded a total of 363 patients with mild (n = 250, 68.9%), moderate (n = 94, 25.9%) and severe (n = 19, 5.2%), and Metzer health catchment recorded 273 patients with mild (n = 207, 75.8%), moderate (n = 45, 16.5%), and severe (n = 21, 7.7%) conditions.

### Study design, sampling and participants

A cross-sectional survey of lymphoedema patients and their caregivers was conducted over a three-week period in June 2018. Lymphoedema patients were randomly selected from the available database and aimed to include 30 patients with mild, moderate and severe lymphoedema (total n = 90), which would provide a representative sample for each staging group based on an infinite population size, utilising a 90% confidence level and a 15% margin of error. For bi-lateral lymphoedema patients, the stage of the limb most severely affected was used. The WHO definition of caregiver (19) was used as the study’s operational definition of a caregiver. Based on the operational definition, patients identified their main caregiver (s) (maximum of two individuals) and if eligible, were invited to participate in the survey. In both the lymphoedema patients and patient caregivers’ questionnaires, an ADLA or an acute attack was defined as ‘*an episode of acute inflammation*, *pain*, *redness and swelling in the limb*, *associated with lymph nodes and fever’*.

Two semi-structured questionnaires, one for the lymphoedema patients and one for the patient caregivers, were administered by the field team which comprised one researcher, two field assistants, and a clinician. The clinician confirmed if the patient’s lymphoedema was related to either LF or podoconiosis, assessed the stage of the lymphoedema and elicited information on symptoms related to acute attacks. If the patient or caregiver did not know what an acute attack was, then the clinician would ask if the patient had experienced fever, pain, redness or swelling of their affected limbs. Both questionnaires were translated into Amharic and piloted within the team and conducted in an interview-style. The clinician was fluent in English, Amharic and Arigna (the local dialect), and provided language assistance to participants if required. Data was collected in English using electronic tablets and the Open Data Kit (ODK) software [43].

#### Lymphoedema patients

Patients with lymphoedema related to LF or podoconiosis, aged >18 years of age, and who were able to identify a caregiver were invited to participate in the survey. The patient questionnaire was administered to obtain information on:

- Clinical condition (mild, moderate, severe) including the frequency of ADLA episodes in the past six months (number in total)
- Socio-demographic characteristics (gender, age, marital status, educational attainment)
- Identification of their main caregiver(s) (name, age and relationship to the patient). The information of a maximum of two caregivers per patient was documented.

#### Patient caregivers

Caregivers who were identified by the patients and aged >10 years were invited to participate in the survey and interviewed separately from their patients. The caregiver questionnaire was administrated to obtain information on:

- Socio-demographic characteristics (gender, age, marital status, educational attainment)
- Types of care provided in the absence and presence of an ADLA, which were broadly grouped as ‘symptom management’ (care to relieve patients’ symptoms) and ‘day-to-day assistance’ (care to mitigate patients’ loss of independence). The type of care related to these two groups was further categorised as; one-to-one (requiring direct hands-on assistance), inside the household (indirect care provided inside the household), outside the household (indirect care provided outside the household) and other (summarised in Table 1)
- The time spent providing each reported symptom management and day-to-day assistance task was recorded, both in the absence and presence of an ADLA.
- The QoL questions were adapted from the Lymphatic Filariasis Quality of Life (LFSQQ) questionnaire, which has been validated in previous LF studies [44]. The questionnaire consisted of nine domains, and caregivers ranked them according to a four-point scale (no problem = 1, mild = 2, moderate = 3, severe = 4). The QoL domains included; Planning (had difficulty to plan); Neglect (felt devalued by their community); Health (worried about their health); Future (worried about the future); Romantic relationships (difficulties in new/existing romantic relationships); Social problems inside and outside the house (difficulties in participating in social activities inside or outside their house); Household (difficulties completing their household duties) and work/school (difficulties attending their work or school).
- The impact on caregivers’ productivity (number of days care is provided per month, number of work/school days are forfeited per month)
- Any additional comments provided by the caregivers were summarised as quotes under main themes.

**Table 1:** Symptom management care and day-to-day assistance categorised.

	Symptom management tasks	Day-to-day assistance tasks
**One-to-one**	Washing/drying/massaging/applying creams to the limb (s)	Helping with washing/ dressing / mobility
**Inside the household**	Boiling water to wash the limb	Cooking, washing, laundry, childcare, cleaning
**Outside the household**	Seeking health care, purchasing medicine, collecting water to wash the limb(s)	Selling/buying market goods, collecting water/wood, tending animals, farming
**Other**	Praying, collecting ingredients/providing local remedies, providing money to purchase soap	----

### Data analysis

The survey data was exported to and visualised in Microsoft Excel and statistically analysed in SPSS (version 23)[45]. For all statistical tests, a p-value ≤0.05 was considered statistically significant.

To compare the socio-demographic information between different lymphoedema severity groups and their caregiver(s), the Chi-squared test was used. The mean and the 95% CI were calculated for the reported frequency of ADLAs in the last six months, number of days that care was provided per month and the number of work/school days forfeited per month. The symptom management and day-to-day assistance tasks were categorised and compared using the Chi squared test, both in the absence and presence of an ADLA. Further, the types of tasks performed by female and male caregivers were compared using the Chi squared test. The time mild/moderate and severe patient caregivers spent providing each type of care was summed within their respective categories. For each category, the mean time and the 95% CI was calculated and compared in the absence and presence of an ADLA with a Wilcoxon signed-rank test. When comparing the gender of the caregiver and the time spent per task category, a Mann Whitney U test was used.

For the QoL, the overall score was quantified by summing the scores allocated to each domain question, based on the following answer scores; no problem = 1, mild = 2, moderate = 3, severe = 4 [39]. The minimum-maximum overall QoL score per person ranged from 9–36. The QoL mean scores (overall and per domain) of mild/moderate and severe patient caregivers were compared both in the absence and presence of an ADLA with a Mann-Whitney U test.

The additional qualitative comments from caregivers were grouped into themes exploring; the types of care provided (social expectations, demanding aspects of care), impact on their productivity (loss of income, impact on children) and on their QoL (relationship with the patient and family members, social withdrawal, afflicted stigma and psychological stress).

## Results

### Lymphoedema patients

#### Clinical conditions

A total of 73 lymphoedema patients participated in the survey and had mild (n = 21, 28.8%), moderate (n = 21, 28.8%) and severe (n = 31, 42.5%) conditions verified. Most patients had bi-lateral lymphoedema (n = 69, 94.5%). Due to limitations in obtaining adequate numbers of lymphoedema patients with mild and moderate conditions as patients had moved away, died or were not available, these two lymphoedema stages were combined to form a mild/moderate group (n = 42, 57.5%), which was compared to the severe group (n = 31, 42.5%) in the analyses. All patients reported experiencing an ADLA in the past six months. On average, mild/moderate patients reported 6.1 (CI± 2.18) ADLAs, and severe patients 9.8 (CI± 3.17) ADLAs in the last six months.

#### Socio-demographic characteristics

A total of 48 female (65.8%) and 25 male (34.2%) lymphoedema patients participated in the survey. Patients’ mean age was 47.7 years (female 49.2 years (CI±4.88); male 44.8 years (CI±8.80)) and ranged from 20–90 years. Approximately half the patients were married (n = 39, 53%), with one fifth divorced (n = 15, 20.5%) or widowed (n = 15, 20.5%). Most patients were illiterate with no school education (n = 54, 74%). Overall, there was no significant difference in socio-demographic characteristics between the mild/moderate and severe patient group, except by gender, where a higher proportion of female patients had mild/moderate condition than severe, and a higher proportion of male patients had severe than mild/moderate (p = 0.29) (S1 Table).

#### Main caregivers

**Fig 1:**
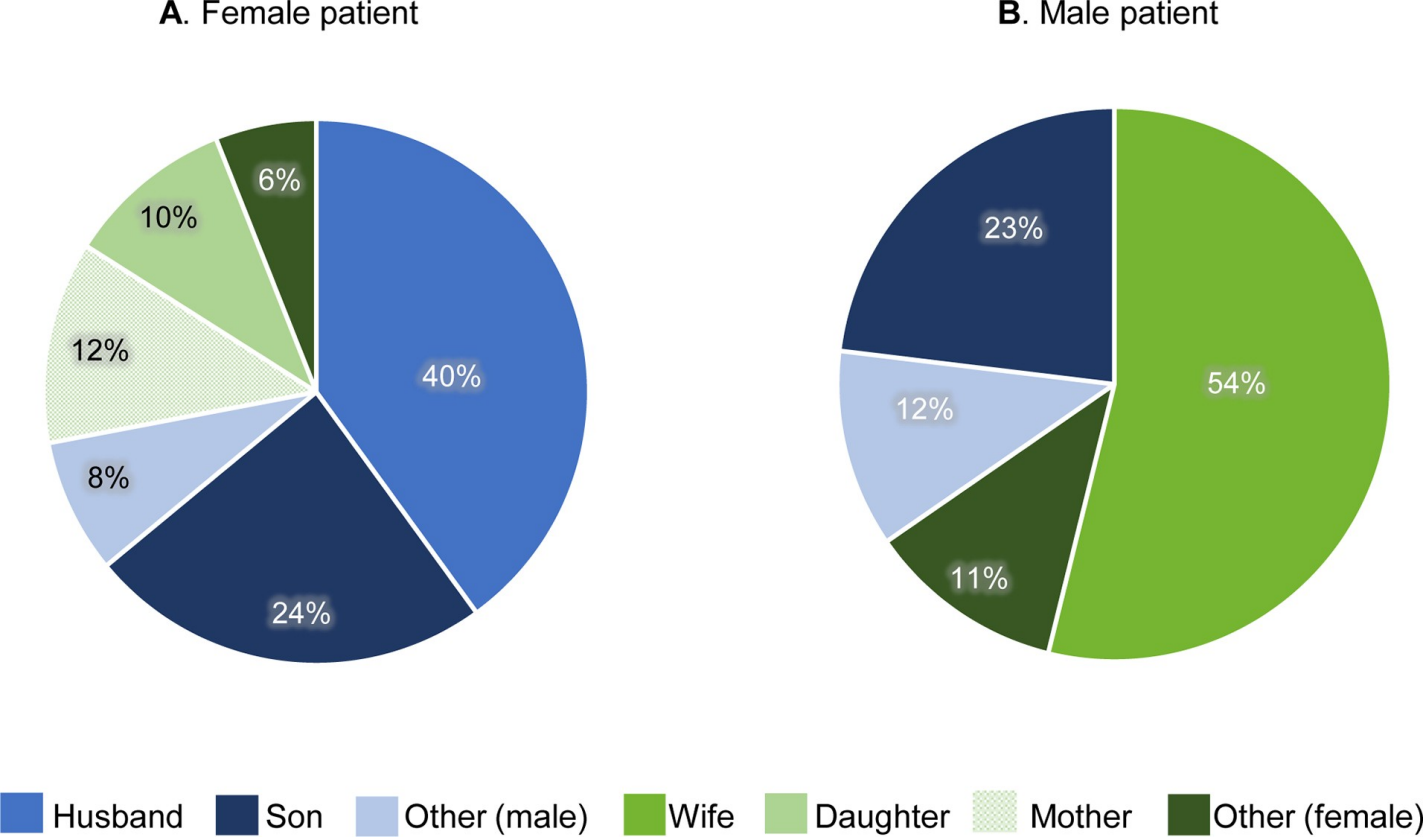
The relationship between male and female patients and their caregivers.

A total of 76 caregivers were identified, of which 45 (59.2%) provided care to mild/moderate patients and 31 (40.8%) provided care to severe patients. Three patients reported having two caregivers. Spouses formed the largest caregiving group for both female and male patients as shown in Fig 1A and 1B with 40% (n = 20) of female patients being cared for by their husband, and 54% of male patients by their wife (n = 14). Sons were the second main caregiver group for both female (n = 12, 24%) and male (n = 6, 23%) patients. Other caregivers included daughters, church leaders, neighbours, sons-in-law and nieces.

### Patient caregivers

#### Socio-demographic information

Of the 76 caregivers interviewed, 31 (40.8%) were female and 45 (59.2%) were male. Caregivers’ mean age was 37.6 years (female 38 years (CI±5.95); male 37.3 years (CI±4.76)) and ranged from 13–80 years. Three caregivers were children aged 13, 15 and 17 years. The majority of caregivers were married (n = 61, 80.3%) and illiterate with no schooling (n = 48, 63.2%). Overall, there was no significant difference in socio-demographic characteristics between mild/moderate and severe patient caregivers (S2 Table). One severe patient caregiver reported that their patient did not experience ADLAs, and hence was not included in the analysis.

#### Types of care

The two types of care and tasks that patient caregivers provided in the absence and in the presence of an ADLA are summarised in Table 2 (A maximum of two tasks were recorded per caregiver). Overall, in the absence of an ADLA, 25 (55.6%) mild/moderate and 17 (56.7%) severe patient caregivers provided no help to manage patients’ symptoms or to assist with their day-to-day activities. However, this reduced significantly in the presence of an ADLA with all, except one caregiver, needing to provide help (Table 2).

**Table 2:** A summary of the types of care and the tasks caregivers provided.

Types of care	Tasks ^*b*^	Mild/moderate (N = 45)					Severe (N = 30) ^*a*^				
		No ADLA		ADLA		p-value	No ADLA		ADLA		p-value
		n	%	n	%		n	%	n	%	
**Symptom management**	**No help**	33	73.3	4	8.9	0.000*	21	70.0	5	16.7	0.000*
	**One-to-one**	5	11.1	14	31.1	0.020*	0	0.0	7	23.3	0.005*
	**Inside**	1	2.2	1	2.2	1.000	6	20.0	6	20.0	1.000
	**Outside**	5	11.1	32	71.1	0.000*	2	6.7	19	63.3	0.000*
	**Other**	2	4.4	6	13.3	0.138	2	6.7	3	10.0	0.640
**Day-to-day assistance**	**No help**	31	68.9	7	15.6	0.000*	24	80.0	7	23.3	0.000*
	**One-to-one**	2	4.4	3	6.7	0.645	0	0.0	1	3.3	0.313
	**Inside**	6	13.3	18	40.0	0.004*	3	10.0	12	40.0	0.007*
	**Outside**	10	22.2	26	57.8	0.001*	5	16.7	20	66.7	0.000*
**Overall no help**		25	55.6	1	2.2	0.000*	17	56.7	0	0.00	0.000*

* p≤0.05

^**a**^ One caregiver was removed from the analysis as their patient did not suffer from ADLA episodes

^**b**^ A maximum of two tasks were recorded per caregiver

#### Symptom management

**Fig 2:**
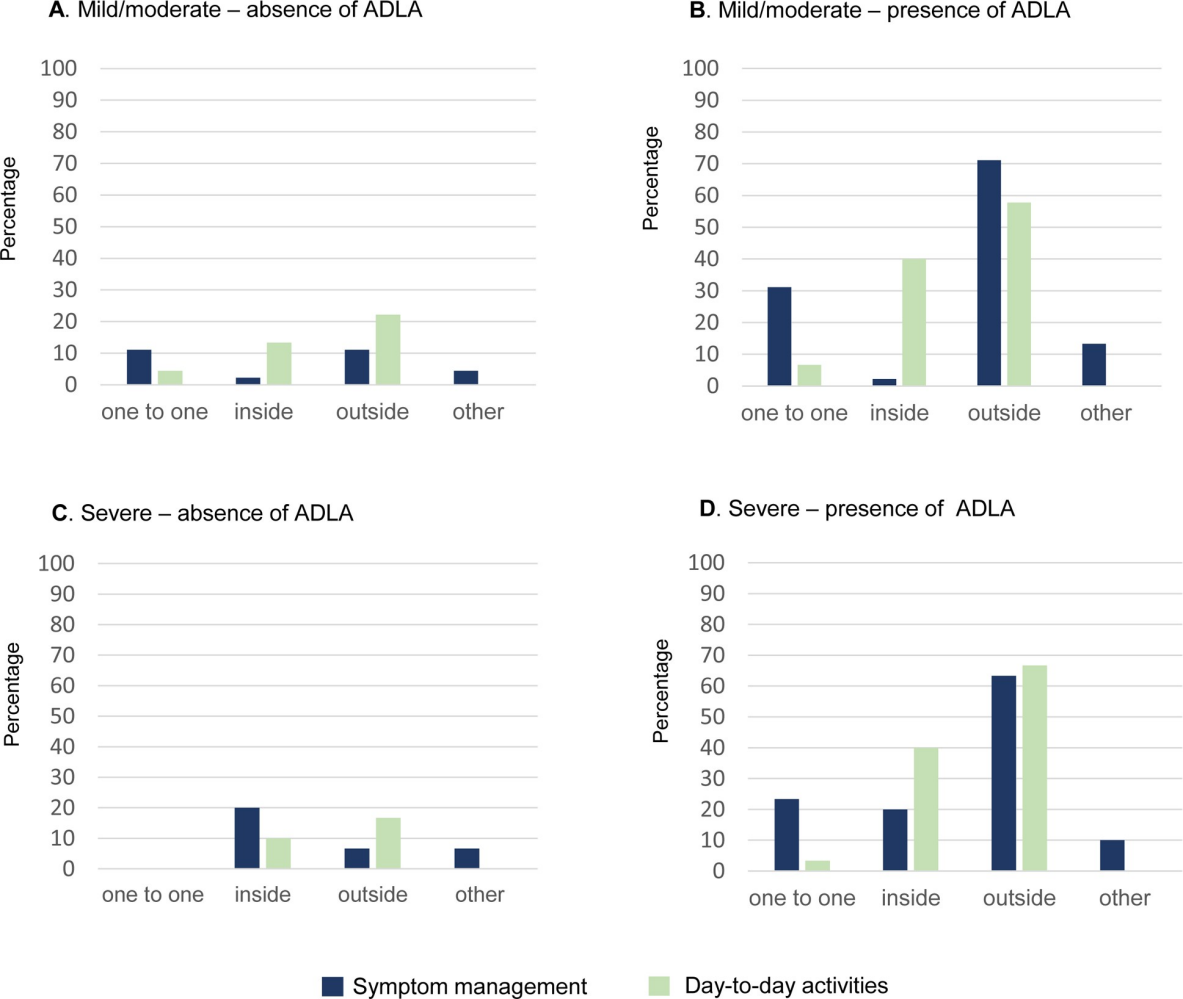
Comparison of the types of care provided by the mild/moderate and severe patient caregivers in the absence and presence of an ADLA.

For symptom management care, in the absence of an ADLA the majority of mild/moderate (n = 33, 73%) and severe (n = 21, 70%) patient caregivers provided no help. However, this changed significantly in the presence of an ADLA as shown in Table 2. There was a significant increase in patient caregivers providing help one-to-one and outside the house for both the mild/moderate and severe patients. This significant change is illustrated in Fig 2A–2D. In the presence of an ADLA, 18 caregivers (mild/moderate n = 12; severe: n = 6) performed multiple tasks. There was no significant difference in the types of care and the tasks provided by the gender of the patient caregiver. However, a higher proportion of female caregivers provided support with one-to-one tasks (mild/moderate: 46.7%; severe: 33.3%) than male caregivers (mild/moderate: 23.3%; severe: 13.3%) (Table 3).

**Table 3:** A summary of the types of care and the tasks provided by the gender of the caregiver in the presence of an ADLA.

Types of care	Tasks ^*b*^	Mild/moderate (N = 45)					Severe (N = 30) ^*a*^				
		Female (N = 15)		Male (N = 30)		p-value	Female (N = 15)		Male (N = 15)		p-value
		n	%	n	%		n	%	n	%	
**Symptom management**	**One to one**	7	46.7	7	23.3	0.111	5	33.3	2	13.3	0.195
	**Inside**	1	6.7	0	0.00	0.153	5	33.3	1	6.7	0.068
	**Outside**	10	66.7	22	73.3	0.642	9	60.0	10	66.7	0.705
	**Other**	0	0.00	6	20.0	0.063	1	6.7	2	13.3	0.543
**Day-to-day assistance**	**One to one**	2	13.3	1	3.3	0.205	1	6.7	0	0.00	0.309
	**Inside**	6	40.0	12	40.0	1.000	7	46.7	5	33.3	0.456
	**Outside**	5	33.3	21	70.0	0.019	10	66.7	10	66.7	1.000

^**a**^ One caregiver was removed from the analysis as their patient did not suffer from ADLA episodes

^**b**^ A maximum of two tasks were recorded per caregiver

#### Day-to-day assistance

For day-to-day assistance, in the absence of an ADLA, the majority of mild/moderate (68.8%) and severe (80%) patient caregivers provided no help. However, this changed significantly in the presence of an ADLA as shown in Table 2. There was a significant increase in patient caregivers providing help inside and outside the house for both the mild/moderate and severe patients. This significant change is illustrated in Fig 2A–2D. In the presence of an ADLA, 42 caregivers (mild/moderate n = 27; severe: n = 15) performed multiple tasks. There was no significant difference in the types of care and the tasks provided by the gender of the patient caregiver. However, a higher proportion of female caregivers provided support with one-to-one tasks than male caregivers (Table 3).

#### Caregiving time

Overall, in the absence of an ADLA, mild/moderate (mean: 00:17 (hours (hr):minutes (min)), CI = ±00:08 (hr:min) and severe (mean: 00:10 (hr:min), CI = ±00:07 (hr:min)) patient caregivers spent little time in providing help to manage patient’s symptoms and assist their day-to-day activities. However, this significantly increased in the presence of an ADLA (mild/moderate mean: 00:47 (hr:min), CI = ±00:11(hr:min); severe mean: 00:51 (hr:min), CI = ±00:16 (hr:min)). During an ADLA, all patient caregivers increased the amount of time per task to help manage patients’ care.

#### Symptom management

For symptom management care, in the absence of an ADLA, patient caregivers spent the longest time providing help for tasks outside the household mild/moderate: mean = 00:11 (hr:min), CI: ±00:12 (hr:min); severe: mean = 00:04 (hr:min), CI: ±00:06 (hr:min)), which increased significantly in the presence of an ADLA (mild/moderate: mean = 01:46 (hr:min), CI: ±00:12 (hr:min); severe: mean = 01:31 (hr:min), CI: ±00:40 (hr:min)) (Table 4). There was no significant difference in the types of care and the tasks provided by the gender of the patient caregiver. However, a higher proportion of female caregivers spent more time providing care for one-to-one tasks than male caregivers (Table 5). 

**Table 4:** A summary of the mean time spent per category (hr: min) in both types of care, both in the absence and presence of an ADLA.

Types of care	Tasks ^*b*^	Mild/moderate (N = 45)					Severe (N = 30) ^*a*^				
		No ADLA		ADLA		p-value	No ADLA		ADLA		p-value
		Time (hr:min)	± CI	Time (hr:min)	± CI		Time (hr:min)	± CI	Time (hr:min)	± CI	
**Symptom management**	**One-to-one**	00:03	00:03	00:08	00:05	0.038*	00:00	00:00	00:09	00:07	0.016*
	**Inside**	00:01	00:01	00:01	00:01	1.000	00:04	00:03	00:04	00:03	1.000
	**Outside**	00:11	00:12	01:46	00:40	0.000*	00:04	00:06	01:31	00:40	0.000*
	**Other**	00:06	00:11	00:12	00:13	0.059	00:01	00:01	00:03	00:05	0.180
	**Total**	00:05	00:04	00:32	00:12	0.000*	00:02	00:02	00:27	00:12	0.000*
**Day-to-day assistance**	**One-to-one**	00:01	00:02	00:04	00:07	0.285*	00:00	00:00	00:02	00:04	0.317
	**Inside**	00:27	00:23	01:08	00:34	0.014*	00:21	00:24	01:06	00:42	0.073
	**Outside**	01:13	00:49	02:06	00:50	0.012*	00:39	00:44	03:02	01:22	0.009*
	**Total**	00:34	01:13	01:06	01:01	0.000*	00:20	00:59	01:23	01:16	0.001*
**Overall mean time spent**		00:17	00:08	00:47	00:11	0.000*	00:10	00:07	00:51	00:16	0.000*

* p≤0.05

^**a**^ One caregiver was removed from the analysis as their patient did not suffer from ADLA episodes

^**b**^ The time of a maximum of two tasks were recorded per caregiver

**Table 5:** A summary of the mean time each spent per task category (hr: min) in both types of care during an ADLA.

Types of care	Tasks ^*b*^	Mild/moderate (N = 45)					Severe (N = 30) ^*a*^				
		Female (N = 15)		Male (N = 30)		p-value	Female (N = 15)		Male (N = 15)		p-value
		Time (hr:min)	± CI	Time (hr:min)	± CI		Time (hr:min)	± CI	Time (hr:min)	± CI	
**Symptom management**	**One to one**	00:13	00:12	00:06	00:05	0.112	00:11	00:10	00:06	00:09	0.250
	**Inside**	00:03	00:06	00:00	00:00	0.157	00:07	00:06	00:01	00:03	0.080
	**Outside**	01:26	01:12	01:58	00:54	0.509	01:15	00:50	01:48	01:07	0.536
	**Other**	00:00	00:00	00:18	00:19	0.067	00:04	00:09	00:03	00:04	0.605
	**Total**	00:24	00:16	00:35	00:16	0.897	00:24	00:14	00:29	00:19	0.476
**Day-to-day assistance**	**One to one**	00:13	00:28	00:00	00:01	0.191	00:04	00:09	00:00	00:00	0.317
	**Inside**	00:58	01:10	01:01	00:41	0.663	01:02	00:43	01:09	01:19	0.574
	**Outside**	00:50	01:18	02:30	01:06	0.055	02:35	01:53	03:29	02:12	0.641
	**Total**	00:57	00:33	01:10	00:28	0.399	01:14	00:42	01:33	00:54	0.772
**Overall mean time spent**		00:39	00:17	00:50	00:15	0.470	00:45	00:20	00:57	00:25	0.440

*p≤0.05

^**a**^ One caregiver was removed from the analysis as their patient did not suffer from ADLA episodes

^**b**^ The time of a maximum of two tasks were recorded per caregiver

#### Day-to-day assistance

For day-to-day assistance, in the absence of an ADLA, patient caregivers spent the longest time providing help for tasks outside the household (mild/moderate: mean = 00:13 (hr:min), CI: ±00:49 (hr:min); severe: mean = 00:39 (hr:min), CI: ±00:44 (hr:min)), which increased significantly in the presence of an ADLA (mild/moderate: mean = 02:35 (hr:min), CI: ±01:53 (hr:min); severe: mean = 03:29 (hr:min), CI: ±02:12 (hr:min)) (Table 4). There was no significant difference in the types of care and the tasks provided by the gender of the patient caregiver. However, a higher proportion of female caregivers spent more time providing care for one-to-one tasks than male caregivers (Table 5).

### Quality of Life

Overall in the absence of an ADLA, the overall mean QoL score (mild/moderate: score = 1.4; severe: score = 1.4) and the mean QoL score for each domain (mild/moderate range = 1.1–1.7, severe range = 1.2–1.5) were low, reflecting no or mild problems in caregiving (Table 6). However, this changed in the presence of an ADLA, and the overall mean QoL score (mild/moderate: score = 2.8; severe: score = 2.8) and the mean QoL score for each domain (mild/moderate range = 1.9–3.6, severe range = 1.7–3.6) increased significantly, reflecting moderate to severe problems in caregiving. For each of the domains, the mean QoL score was low in the absence of an ADLA and significantly increased in the presence of an ADLA. The domains; supporting the household and attending work/school, observed the greatest difference in the mean QoL score between the absence and the presence of an ADLA.

**Table 6:** A summary of caregivers’ mean score per QoL domain.

Domains	Mild/moderate (N = 45)			Severe (N = 30) ^*a*^		
	QoL scores ^b^			QoL scores ^b^		
	No ADLA	ADLA	p-value	No ADLA	ADLA	p-value
**Plans**	1.4	3.0	0.000*	1.2	3.1	0.000*
**Neglect**	1.7	1.9	0.387	1.5	2.0	0.260
**Future**	1.6	2.5	0.005*	1.5	2.5	0.000*
**Health**	1.5	2.4	0.017*	1.5	2.5	0.110
**Romantic relationships**	1.1	1.9	0.003*	1.3	1.7	0.363
**Social outside**	1.1	3.1	0.000*	1.1	2.8	0.000*
**Social inside**	1.2	3.2	0.000*	1.2	3.0	0.000*
**Household**	1.4	3.5	0.000*	1.5	3.6	0.000*
**Work/school**	1.4	3.6	0.000*	1.4	3.6	0.000*
**Mean of overall score**	1.4	2.8	0.000*	1.4	2.8	0.000*

*p≤0.05

^**a**^ One caregiver was removed from the analysis as their patient did not suffer from ADLA episodes

^**b**^ Category defined by the range of the mean QoL Score per domain: No problem: 1–1.4; Mild: 1.5–2.5; Moderate: 2.5–3.4; Severe: 3.5–4

**Fig 3:**
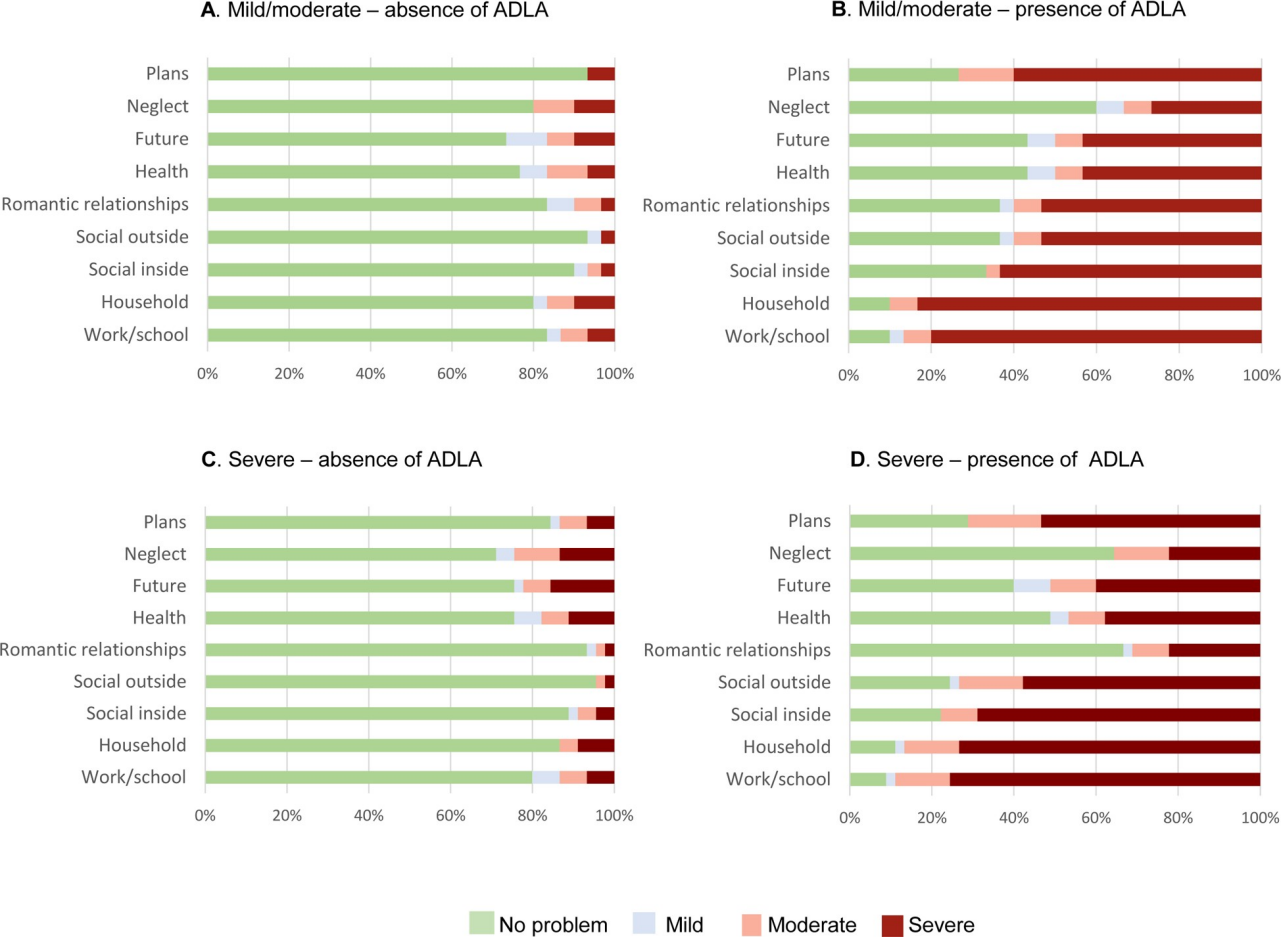
Percentage of QoL responses provided by the mild/moderate and severe patient caregivers in the absence and presence of an ADLA.

The changes in QoL across the nine domains are illustrated in Fig 3A and 3B for mild/moderate patient caregivers and Fig C-D for severe patient caregivers. The proportion of caregivers reporting a severe problem with their QoL ranged from 1–7% in the absence of an ADLA (mild/moderate range 1–3%; severe 1–7%) which then increased significantly to 8–34% (mild/moderate 8–25%; severe 10–34%).

### Impact on productivity

Per month, mild/moderate and severe patient caregivers provided care on average for 14.8 (CI ±3.31) and 15.5 (CI ±4.33) days respectively. No significant difference was found between the severity groups (p = 0.813). Per month, caregivers forfeited a similar amount of work/school days (mild/moderate: 7.17; (CI ±1.92); severe 5.87; (CI ±1.89)).

### Additional comments

There were three main themes related to the additional comments that caregivers provided and included i) types of care ii) impact on productivity and iii) QoL

#### Types of care

*Social expectations*: Two male caregivers reported that socially constructed gender norms acted as a barrier when providing certain types of care.

*‘The culture prevents me to help my wife…’* (Male, 60)

*‘Many do not understand the caring duties I have’* (Male, 28)

*Demands of caregiving*: Many caregivers (n = 14) reported challenges related to their poverty.

*‘I want to take her to the clinic*, *but I am too poor’* (Male, 70)

*‘Her situation and my poverty stress me out*. *I do not know how to help’* (Male, 29)

#### Impact on productivity

*Loss of income*: Many caregivers reported financial problems (n = 35) and decreased food security (n = 7).

*‘We do not have enough money for food’* (Male, 28)

*‘During her acute attack she stays in bed for 7 days*, *so I stay with her and lose 7 days of farming*. *Our family unit is becoming very poor’* (Male, 29)

*Impact on children*: Many caregivers (n = 11) reported not being able to send their children to school.

*‘The children also suffer… unable to go to school as we have no money for the uniforms’* (Male, 60)

*‘When I see my friends*, *they are all in eighth grade*. *I worry about my future’* (Female, 15)

#### Quality of life

*Relationship with the patient*: Many found caregiving rewarding (n = 26), however, many also reported their relationship with the patient to be deteriorating, notably during ADLAs (n = 14).

*‘We exchange many blessings’* (Female, 65)

*‘I feel like I have lost my husband’* (Female, 60)

*Relationship with other family members*: Two caregivers reported that their relationship with other family members had been impacted.

*‘I have separated from my husband to provide care to my mother’* (Female, 35)

*‘I feel like I have lost my ‘normal wife’* (Male, 36)

*Social withdrawal*: Many caregivers (n = 29) reported that they had no time to socialise with friends or to contribute to community activities.

*‘Many (friends) do not understand the caring duties I have’* (Male, 28)

*‘As an Ethiopian we are encouraged to participate in many social activities*, *the community life is very important to me’* (Female, 37)

*Afflicted stigma*: Two caregivers reported being victim of afflicted stigma or discrimination.

*‘…my mother’s condition is contagious*, *and people avoid me*.*’* (Male, 29)

*‘…why God has placed such an illness on our family’* (Male, 19)

*Psychological stress*: Many (n = 9) suffered seeing their relative suffer, and two caregivers felt unable to cope with the patients’ suicidal thoughts during ADLAs.

*‘During an acute attack my wife gets suicidal thoughts*. *I am scared to leave her alone’* (Male, 28)

*‘…if I do not provide enough care*, *she threatens she will commit suicide’* (Male, 65)

## Discussion

Lymphoedema patients were found to be predominately female, middle aged and illiterate, which aligns with LF studies elsewhere [28,29], and podoconiosis studies conducted in similar Ethiopian settings [16,46]. Patients predominately had bi-lateral limb lymphoedema, which is largely associated with podoconiosis related lymphoedema [12]. This present study did not differentiate between the aetiology of the lymphoedema as it sought to quantify the impact of providing care to patients suffering from both LF and podoconiosis related lymphoedema. However, differential diagnosis may be important in certain circumstances, and previous studies have utilised tests such as filarial antigen testing, filarial antibody testing and parasitological examination to exclude an LF diagnosis in LF and podoconiosis co-endemic areas [15].

All patients experienced frequent ADLAs, with a higher frequency of ADLAs in patients with severe stage lymphoedema [6,7,42], which contribute to the progression of disease and are key predictors of disability [8–10]. Whilst recall bias may have influenced the number of ADLAs reported, patients with mild/moderate and severe lymphoedema experienced an average of 6 and 10 respectively, which may be broadly extrapolated to 12 to 20 ADLAs per year. This aligns with the findings of a recent paper, which documented that podoconiosis patients with persistent lymphoedema experienced an average of 22 ADLA episodes a year [47]. Regardless of the lymphoedema severity, whilst experiencing an ADLA, patients’ increased reliance on informal care, from their caregivers, to manage their symptoms and day-to-day activities was observed.

Caregivers were found to be predominately male, middle aged, illiterate and a family member of the patient. The caregivers’ age and illiteracy in this study aligns with previous LMIC caregiving research, but wives and daughters were more often identified as LMIC caregivers [20,48]. However, several caregiving studies have identified that patients’ spouses often became their caregivers [23], which may explain this study’s higher proportion of male caregivers and lack of daughter caregivers providing care to male patients. Although this study’s small sample size limits the generalisability, the gender and role of caregivers is likely to be determined by the patient’s disease, gender and care needs, which may vary greatly by the clinical nature of the disease, cultural and social factors, and epidemiological distribution. This is currently not well understood in the context of NTDs and more research on different diseases in different settings are required to obtain a more comprehensive picture and to monitor equity [33].

Caring for patients during ADLAs was found to have a significant impact on caregivers, who needed to provide more support for symptom management and day-to-day tasks, including help with one-to-one tasks, such as limb washing. This is important as the WHO recommends regular limb hygiene to mitigate the morbidity associated with lymphoedema and enabling the continuity of such tasks during an ADLA, demonstrates caregivers’ indispensable role as a human resource in providing the MMDP programmes’ minimum package of care [10,12,47]. In addition, tasks outside the household, which often mitigated the household’s economic loss, such as farming or tending the animals, were prominently provided by caregivers to assist patients’ day-to-day activities during an ADLA.

Our study found some differences by gender in the types of day-to-day care tasks provided. Tasks inside the household, such as cooking, and one-to-one care, were more often performed by female caregivers. Studies in comparable settings have documented similar caregiving tasks and related to traditional feminine role [49,50]. Tasks outside the household were more often performed by male caregivers. Understanding the different gender roles in caregiving may help to achieve the SDG 5, target 5.4, which seeks to value informal domestic work [36]. Albeit, it was challenging to distinguish between activities which females were ‘expected’ to perform on a routine basis [26] and those which were executed in their capacity as a caregiver.

Patients’ productivity during ADLAs and its contribution to their household’s economic downfall has been documented [17,51]. However, our research helps to uncover and quantify a hidden aspect to the economic burden that family units suffer and suggests that previous evaluations have grossly underestimated the magnitude of the burden that lymphoedema poses on households’ economy. The hardship experienced by family units is not only due to the patients decreased working hours, but, in union with caregivers’ lack of productivity whilst providing care. Similarly, a study exploring the impact of ADLAs on podoconiosis patient caregivers in Ethiopia, reported that, at times, caregivers forfeited work to care for their relative [31]. Although there may be recall bias in remembering the frequency and impact of ADLAs, studies in Tanzania [52] and Malawi [29] have reported that patients lose between three and eleven days per ADLA episode, which aligns with our study where caregivers reported losing an average of six to seven work or school days per month. Forfeiting productive hours, often minimises time to farm and curtails household profit that can strain food security [32].

Caregiving inflicts a multitude of stressors, which collectively contribute to a decreased QoL [24]. We found that when patients experienced an ADLA, the caregivers’ QoL was negatively impacted. Notably, caregivers found more difficulties with participating in social activities inside or outside their house, completing their household duties and attending their work or school. The impact on socialising may be due to time constraints or socially constructed barriers, such as stigma linked to the diseases [26] or to the gender-bias largely associated with caregiving [49]. The impact on child caregivers not attending school is of a particular concern with long-term implications, which urgently need to be addressed.

This study reveals the concerning state of LF and podoconiosis patient caregivers, a previously hidden dimension to the public health burden posed by NTDs, which identifies the need for accessible support services. Caregivers’ role in achieving the global aims of eliminating and mitigating the disability caused by these diseases needs to be better understood and supported. The study highlights the need to scale-up effective MMDP programmes to reduce the overall burden of disease and occurrence of ADLAs, thereby, alleviating the suffering of patients and reducing the impact on their caregivers, which will also help to address the SDG targets.

## Supporting information

S1 STROBE Checklist (DOC)

S1 Table A comparison of mild/moderate and severe patients’ socio-demographic information. (DOCX)

S2 Table A comparison of mild/moderate and severe patient caregivers’ socio-demographic information. (DOCX)
