# Supplementary material for: Quantifying the socio-economic impact of leg lymphoedema on patient caregivers in a lymphatic filariasis and podoconiosis co-endemic district of Ethiopia
Source: PLoS Negl Trop Dis. 2020 Mar 3;14(3):e0008058. doi: 10.1371/journal.pntd.0008058 (PMC7069637; doi:10.1371/journal.pntd.0008058)
Supplement: S1 STROBE Checklist — (DOC) [file pntd.0008058.s001.doc]

STROBE Statement—Checklist of items that should be included in reports of ***cross-sectional studies***

|  | Item No | Recommendation |
| --- | --- | --- |
| **Title and abstract** | 1 | (*a*) Indicate the study’s design with a commonly used term in the title or the abstract  Abstract (paragraph 2 – methodology/principle findings) |
|  |  | (*b*) Provide in the abstract an informative and balanced summary of what was done and what was found  Abstract (paragraph 2 – methodology/principle findings) |
| Introduction | | |
| Background/rationale | 2 | Explain the scientific background and rationale for the investigation being reported  Introduction (paragraph 1, 2, 3, 4, 5) |
| Objectives | 3 | State specific objectives, including any prespecified hypotheses  Introduction (paragraph 6 - line 137 – 139) |
| Methods | | |
| Study design | 4 | Present key elements of study design early in the paper  Methods section – ‘study design, sampling and participants’ |
| Setting | 5 | Describe the setting, locations, and relevant dates, including periods of recruitment, exposure, follow-up, and data collection  Methods section – ‘study site characteristics’ |
| Participants | 6 | *(*a) Give the eligibility criteria, and the sources and methods of selection of participants  Methods section – ‘study design, sampling and participants’ |
| Variables | 7 | Clearly define all outcomes, exposures, predictors, potential confounders, and effect modifiers. Give diagnostic criteria, if applicable  Methods section – ‘study design, sampling and participants’ |
| Data sources/ measurement | 8* | For each variable of interest, give sources of data and details of methods of assessment (measurement). Describe comparability of assessment methods if there is more than one group  Methods section – ‘study design, sampling and participants’ & ‘data analysis’ |
| Bias | 9 | Describe any efforts to address potential sources of bias  Bias reduced by piloting measuring tool – Methods section - ‘study design, sampling and participants’ line 174 |
| Study size | 10 | Explain how the study size was arrived at  Methods section – ‘study design, sampling and participants’ line 166-168 |
| Quantitative variables | 11 | Explain how quantitative variables were handled in the analyses. If applicable, describe which groupings were chosen and why  Methods section – ‘data analysis’ |
| Statistical methods | 12 | *(*a) Describe all statistical methods, including those used to control for confounding  Methods section – ‘data analysis’ |
|  |  | (*b*) Describe any methods used to examine subgroups and interactions  Methods section – ‘data analysis’ |
|  |  | (*c*) Explain how missing data were addressed  N/A |
|  |  | (*d*) If applicable, describe analytical methods taking account of sampling strategy  N/A |
|  |  | (*e*) Describe any sensitivity analyses  N/A |
| Results | | |
| Participants | 13* | (a) Report numbers of individuals at each stage of study—eg numbers potentially eligible, examined for eligibility, confirmed eligible, included in the study, completing follow-up, and analysed  Results section – ‘lymphoedema patients’ and ‘patient caregivers’ |
|  |  | (b) Give reasons for non-participation at each stage  N/A |
|  |  | (c) Consider use of a flow diagram  N/A |
| Descriptive data | 14* | (a) Give characteristics of study participants (eg demographic, clinical, social) and information on exposures and potential confounders  Results section – ‘lymphoedema patients’ and ‘patient caregivers’ – highlighted in Table 1 |
|  |  | (b) Indicate number of participants with missing data for each variable of interest  N/A |
| Outcome data | 15* | Report numbers of outcome events or summary measures  Results section – ‘lymphoedema patients’ and ‘patient caregivers’ |
| Main results | 16 | *(*a) Give unadjusted estimates and, if applicable, confounder-adjusted estimates and their precision (eg, 95% confidence interval). Make clear which confounders were adjusted for and why they were included  Provided within results section. ‘Severity of leg lymphoedema’ – line 260-261 |
|  |  | (*b*) Report category boundaries when continuous variables were categorized  Reported within tables i.e. age categories shown in Table 1 |
|  |  | (*c*) If relevant, consider translating estimates of relative risk into absolute risk for a meaningful time period  N/A |
| Other analyses | 17 | Report other analyses done—eg analyses of subgroups and interactions, and sensitivity analyses  Analyses by stratifying by age groups, sex, severity and in the absence and presence of acute attacks (Table 1, 2, 3, 4, 5, 6) |
| Discussion | | |
| Key results | 18 | Summarise key results with reference to study objectives  Discussion section – paragraph 1, 2, 3, 5, 6) |
| Limitations | 19 | Discuss limitations of the study, taking into account sources of potential bias or imprecision. Discuss both direction and magnitude of any potential bias  Discussion section – paragraph 2 – lines 458-462 |
| Interpretation | 20 | Give a cautious overall interpretation of results considering objectives, limitations, multiplicity of analyses, results from similar studies, and other relevant evidence  Discussion section – paragraph 4 lines 476-478 |
| Generalisability | 21 | Discuss the generalisability (external validity) of the study results  Discussion section – paragraph 2- lines 458-462 |
| Other information | | |
| Funding | 22 | Give the source of funding and the role of the funders for the present study and, if applicable, for the original study on which the present article is based  Included in ‘funding’ |

*Give information separately for exposed and unexposed groups.

**Note:** An Explanation and Elaboration article discusses each checklist item and gives methodological background and published examples of transparent reporting. The STROBE checklist is best used in conjunction with this article (freely available on the Web sites of PLoS Medicine at http://www.plosmedicine.org/, Annals of Internal Medicine at http://www.annals.org/, and Epidemiology at http://www.epidem.com/). Information on the STROBE Initiative is available at www.strobe-statement.org.
