# Supplementary material for: Quantifying the socio-economic impact of leg lymphoedema on patient caregivers in a lymphatic filariasis and podoconiosis co-endemic district of Ethiopia
Source: PLoS Negl Trop Dis. 2020 Mar 3;14(3):e0008058. doi: 10.1371/journal.pntd.0008058 (PMC7069637; doi:10.1371/journal.pntd.0008058)
Supplement: S1 Table — (DOCX) [file pntd.0008058.s002.docx]

**S1 Table. A comparison of mild/moderate and severe patients’ socio-demographic information**

|  |  | **Mild/moderate (N=42)** | | **Severe**  **(N=31)** | | **P-value** |
| --- | --- | --- | --- | --- | --- | --- |
|  |  | **n** | **%** | **n** | **%** |  |
| **Gender** | Female | 32 | 66.7 | 16 | 33.3 | 0.029* |
|  | Male | 10 | 40.0 | 15 | 60.0 |  |
| **Age** | ≤48 | 24 | 58.5 | 17 | 41.5 | 0.845 |
|  | 49> | 18 | 56.3 | 14 | 43.8 |  |
| **Marital status** | Single | 2 | 50.0 | 2 | 50.0 | 0.439 |
|  | Married | 25 | 64.1 | 14 | 35.9 |  |
|  | Divorced | 6 | 40.0 | 9 | 60.0 |  |
|  | Widowed | 9 | 60.0 | 6 | 40.0 |  |
| **Education** | Illiterate | 31 | 57.4 | 23 | 42.6 | 0.971 |
|  | Primary | 10 | 58.8 | 7 | 41.2 |  |
|  | Secondary and higher | 1 | 50.0 | 1 | 50.0 |  |

**p≤0.05*
