# Supplementary material for: Quantifying the socio-economic impact of leg lymphoedema on patient caregivers in a lymphatic filariasis and podoconiosis co-endemic district of Ethiopia
Source: PLoS Negl Trop Dis. 2020 Mar 3;14(3):e0008058. doi: 10.1371/journal.pntd.0008058 (PMC7069637; doi:10.1371/journal.pntd.0008058)
Supplement: S2 Table — (DOCX) [file pntd.0008058.s003.docx]

S2 Table. A comparison of mild/moderate and severe patient caregivers’ socio-demographic information

|  |  | **Mild/moderate**  **(N=45)** | | **Severe**  **(N=31)** | | **p-value** |
| --- | --- | --- | --- | --- | --- | --- |
|  |  | **n** | **%** | **n** | **%** |  |
| **Gender** | Female | 15 | 48.4 | 16 | 51.6 | 0.111 |
|  | Male | 30 | 66.7 | 15 | 33.3 |  |
| **Age** | ≤38 | 32 | 58.2 | 23 | 41.8 | 0.989 |
|  | 39> | 13 | 61.9 | 8 | 38.1 |  |
| **Marital status** | Single | 4 | 66.7 | 2 | 33.3 | 0.475 |
|  | Married | 35 | 57.4 | 26 | 42.6 |  |
|  | Divorced | 6 | 75.0 | 2 | 25.0 |  |
|  | Widowed | 0 | 0.0 | 1 | 100.0 |  |
| **Education** | Illiterate | 26 | 54.2 | 22 | 45.8 | 0.403 |
|  | Primary | 10 | 62.5 | 6 | 37.5 |  |
|  | Secondary and higher | 9 | 75.0 | 3 | 25.0 |  |
